# Long-Term Proton Pump Inhibitor Therapy and Risk of Gastric Atrophy and Precancerous Mucosal Changes: A Systematic Review

**DOI:** 10.7759/cureus.92323

**Published:** 2025-09-14

**Authors:** Adeel Khalid, Bhavna Singla, Shivam Singla, Sunita Kumawat, Abdullah Abbas, Shaheem Ur Rehman, Prem Chand, Lachhmi Bai, Arif Tajammul

**Affiliations:** 1 Internal Medicine, Kulhudhuffushi Regional Hospital, Kulhudhuffushi, MDV; 2 Internal Medicine, Erie County Medical Center Health Campus, Buffalo, USA; 3 Internal Medicine, TidalHealth Peninsula Regional, Salisbury, USA; 4 Internal Medicine, Hackensack Meridian Ocean Medical Center, Brick, USA; 5 Internal Medicine, Shaikh Zayed Hospital, Lahore, PAK; 6 Internal Medicine, Pakistan Air Force Hospital, Islamabad, PAK; 7 Internal Medicine, City Medical Centre, Kandhkot, PAK; 8 Internal Medicine, Chandka Medical College, Karachi, PAK; 9 Internal Medicine, Services Hospital Lahore, Lahore, PAK

**Keywords:** gastric atrophy, gastroesophageal reflux disease, helicobacter pylori, intestinal metaplasia, long-term therapy, proton pump inhibitors

## Abstract

This systematic review evaluated the association between long-term use of proton pump inhibitors (acid-suppressing medications) and the development of gastric atrophy and related precancerous mucosal changes. Six clinical trials involving a total of more than 1,100 patients with acid-peptic disorders were analyzed, encompassing diverse study designs, follow-up durations ranging from two to five years, and variable *Helicobacter pylori* infection status. Across these studies, long-term proton pump inhibitor therapy alone did not consistently accelerate the progression of gastric atrophy or intestinal metaplasia. Three trials demonstrated stability or regression of mucosal changes following eradication of *H. pylori*, while two reported progression of corpus atrophy or metaplasia in patients with persistent infection; one study found minimal overall histological change regardless of therapy. Some studies noted hypergastrinemia and corpus atrophy during prolonged treatment, but these findings were inconsistent and often influenced by baseline histology and geographic variation. Collectively, the evidence underscores the importance of assessing and managing *H. pylori* infection in patients requiring prolonged acid suppression. The available evidence is limited by the small number of trials, modest sample sizes in some studies, and reliance on older data, highlighting the need for updated large-scale investigations.

## Introduction and background

Proton pump inhibitors (PPIs) are among the most widely prescribed medications worldwide and are considered the mainstay of therapy for acid-related gastrointestinal disorders such as gastroesophageal reflux disease (GERD), peptic ulcer disease, and functional dyspepsia [1]. Their efficacy in suppressing gastric acid secretion has made them a cornerstone of both short-term and long-term management strategies. With the increasing availability of over-the-counter formulations and frequent prescribing for prophylactic indications, the duration of proton pump inhibitor therapy has steadily increased in clinical practice [2,3].

While proton pump inhibitors are generally regarded as safe and well-tolerated in the short term, concerns have been raised about their long-term safety profile. Chronic acid suppression can induce hypergastrinemia, a physiological response that has been implicated in enterochromaffin-like cell hyperplasia, fundic gland polyp development, and progression of mucosal changes [4,5]. The role of Helicobacter pylori infection further complicates this relationship, as the bacterium is known to interact with gastric acid suppression in influencing the distribution and severity of gastritis. In H. pylori-positive patients, prolonged use of proton pump inhibitors may accelerate the development of corpus-predominant gastritis and atrophic changes, which are considered precursors to intestinal metaplasia and gastric neoplasia [6].

Evidence from observational studies has provided conflicting results, with some suggesting an association between chronic proton pump inhibitor use and precancerous gastric lesions, while others report minimal or no risk [7,8]. Randomized and prospective clinical trials remain the most reliable source of evidence in this context, yet these studies are relatively limited and in many cases were conducted more than a decade ago. Despite their age, these trials provide valuable insights into the long-term histological consequences of sustained acid suppression and remain essential for guiding current understanding. The objective of this systematic review is to synthesize the available clinical trial evidence on the relationship between long-term proton pump inhibitor therapy and the development of gastric atrophy and precancerous mucosal changes, with particular attention to the modifying effect of Helicobacter pylori infection. Accordingly, our research question was: Does long-term proton pump inhibitor therapy increase the risk of gastric atrophy and precancerous mucosal changes, and how is this risk influenced by the presence or eradication of H. pylori infection?

## Review

Materials and methods

Protocol and Reporting Standards

This systematic review was conducted in accordance with the Preferred Reporting Items for Systematic Reviews and Meta-Analyses (PRISMA) guidelines [9], ensuring transparency in study selection, data extraction, and synthesis. A protocol outlining the objectives, inclusion criteria, and methodology was developed before initiating the search and was strictly followed throughout the review process. The protocol was not registered in PROSPERO, as the review was initiated and substantially progressed before registration was considered, and the scope was focused on synthesizing older clinical trial data rather than generating new clinical recommendations.

Eligibility Criteria (PICO Framework)

The eligibility criteria were structured according to the PICO (population, intervention, comparison, and outcomes) framework [10]. The population of interest included adult patients receiving long-term proton pump inhibitor therapy for gastroesophageal reflux disease or related acid-peptic disorders. The intervention was defined as prolonged use of any proton pump inhibitor, irrespective of type or dose, with or without concomitant Helicobacter pylori eradication therapy. The comparator group consisted of either patients not receiving eradication therapy, patients undergoing surgical alternatives such as anti-reflux surgery, or H. pylori-negative individuals. The primary outcomes included histologically confirmed gastric atrophy, intestinal metaplasia, and related precancerous mucosal changes. Secondary outcomes included hypergastrinemia, changes in gastric inflammation, and the development of gastric polyps. Only clinical trials, including randomized controlled trials and prospective cohort studies with long-term follow-up, were considered eligible for inclusion. In this review, long-term follow-up was defined as a minimum duration of two years of continuous therapy or observation, as shorter periods were unlikely to capture meaningful histological progression or regression.

Search Strategy

A comprehensive literature search was performed using PubMed, Embase, and the Cochrane Central Register of Controlled Trials (CENTRAL) from inception to the most recent date available. The search strategy combined Medical Subject Headings and free-text terms such as “proton pump inhibitors,” “omeprazole,” “lansoprazole,” “esomeprazole,” “gastric atrophy,” “intestinal metaplasia,” “gastric polyps,” and “Helicobacter pylori.” Boolean operators were applied to refine the results, and the search was restricted to human studies published in English. In addition, reference lists of relevant reviews and all included studies were manually screened to identify further eligible trials. The final search was completed on July 31, 2025.

Study Selection

Two independent reviewers screened all titles and abstracts for relevance, followed by full-text review of potentially eligible articles. Discrepancies were resolved by discussion until consensus was reached. The inclusion process was documented in a PRISMA flow diagram, detailing the number of records identified, screened, excluded, and ultimately included in the review.

Data Extraction and Quality Assessment

Data were extracted into a standardized form that captured key study characteristics including author, year, study design, population characteristics, sample size, H. pylori status, intervention details, comparator groups, outcomes measured, follow-up duration, and key findings. The risk of bias for randomized controlled trials was assessed using the Cochrane Risk of Bias 2.0 tool [11], whereas prospective non-randomized trials were evaluated using the Risk of Bias in Non-randomized Studies of Interventions (ROBINS-I) tool [12]. Risk assessments were independently performed by two reviewers and categorized as low or moderate concern.

Data Synthesis

Given the heterogeneity in study design, populations, outcome definitions, and follow-up durations, a meta-analysis was not feasible. Therefore, a narrative synthesis approach was employed to summarize the evidence. Findings were compared across studies to identify consistencies, discrepancies, and potential explanations, with particular attention to the modifying effect of Helicobacter pylori infection on proton pump inhibitor-associated gastric changes. Observational studies were excluded because they are more prone to bias from confounding, lack of standardized histological endpoints, and variable follow-up protocols, which would have limited comparability with randomized and prospective clinical trials. However, we acknowledge that inclusion of such data in a supplementary analysis may provide additional context, particularly regarding real-world prescribing patterns and longer-term outcomes. This remains a potential area for future work to complement the trial-focused synthesis presented here.

Results

Study Selection Process

**Figure 1:**
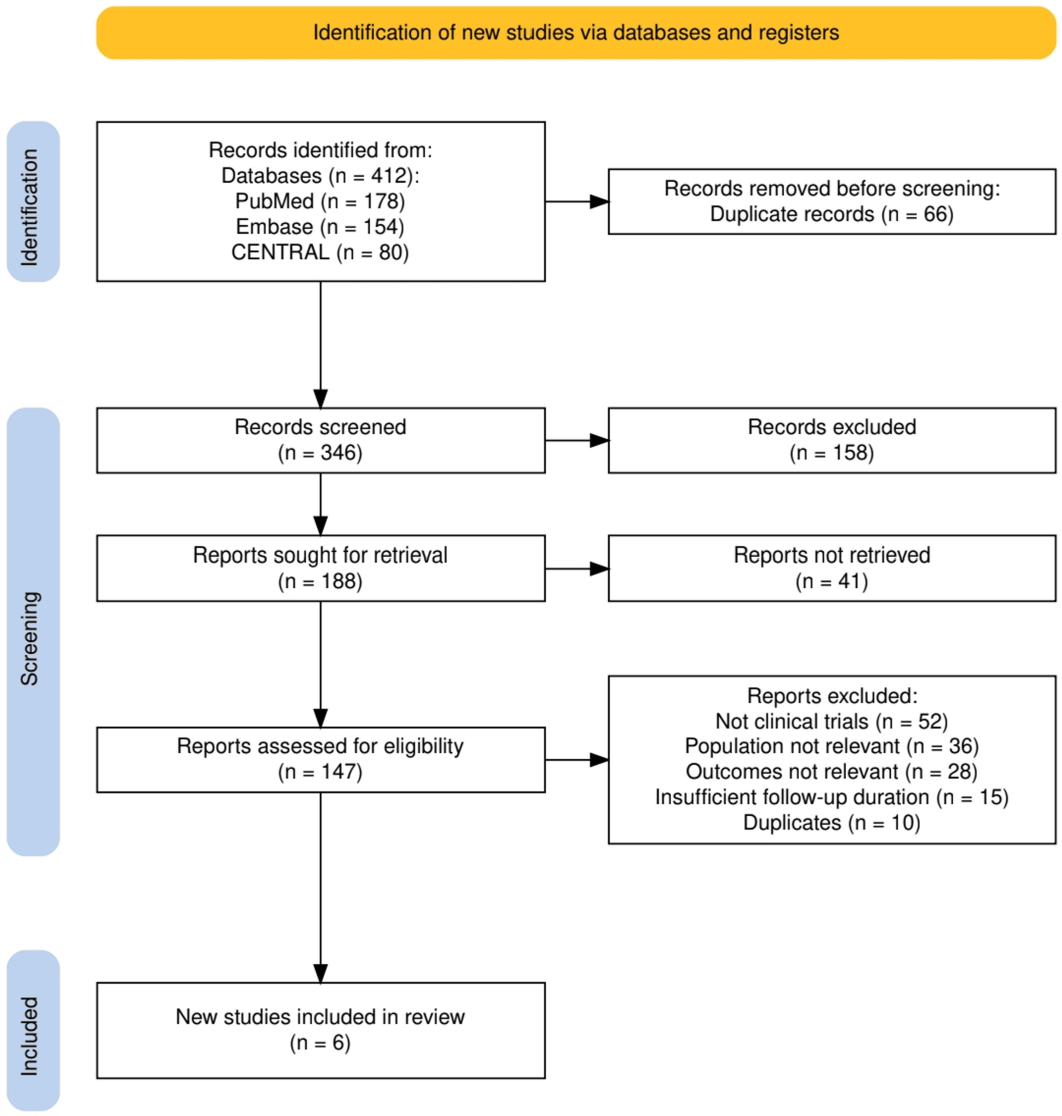
The PRISMA flowchart represents the study selection process. PRISMA: Preferred Reporting Items for Systematic Reviews and Meta-Analyses

The study selection process is illustrated in Figure 1, which outlines the flow of records through the review stages. A total of 412 records were retrieved from PubMed, Embase, and CENTRAL, of which 66 duplicates were removed, leaving 346 unique records for screening. After title and abstract screening, 158 were excluded, and 188 full-text reports were sought, of which 41 could not be retrieved. The remaining 147 articles were assessed for eligibility, and 141 were excluded for reasons including not being clinical trials, irrelevant populations or outcomes, insufficient follow-up, or duplication. Ultimately, six studies met the inclusion criteria and were incorporated into the systematic review.

Characteristics of the Selected Studies

The characteristics of the selected studies are summarized in Table 1, which highlights the diversity of study designs, populations, and follow-up durations. Across randomized controlled trials and prospective clinical studies, patients with gastroesophageal reflux disease or related acid-peptic conditions were evaluated under prolonged proton pump inhibitor therapy, with or without Helicobacter pylori eradication. Interventions varied in terms of PPI type, dosing regimens, and duration, with comparators including surgical approaches, eradication strategies, or H. pylori-negative controls. The primary outcomes consistently focused on histological changes such as gastric atrophy, intestinal metaplasia, and inflammation, while secondary endpoints assessed hypergastrinemia and related biochemical alterations. Collectively, these studies provide a comprehensive framework for examining the interplay between long-term acid suppression, H. pylori infection status, and the risk of precancerous gastric mucosal changes.

**Table 1:** Characteristics of clinical trials evaluating long-term proton pump inhibitor therapy and gastric mucosal changes. GERD: Gastroesophageal reflux disease; PPI: Proton pump inhibitor; ARS: Anti-reflux surgery; IM: Intestinal metaplasia; H. pylori: Helicobacter pylori

Study (Author et al., Year)	Study Design	Population (Sample size, H. pylori status)	Intervention (PPI type, dose, duration)	Comparator	Outcomes Measured (atrophy, intestinal metaplasia, hypergastrinemia, polyps)	Duration of Follow-up	Key Findings
Kuipers et al., 2004 [13]	Randomized controlled trial	231 H. pylori positive GERD patients, on ≥12 months omeprazole	Omeprazole maintenance therapy; subgroup received triple therapy (omeprazole, amoxicillin, clarithromycin for 1 week)	Omeprazole only vs omeprazole + eradication	Gastritis (activity, inflammation, atrophy, intestinal metaplasia)	2 years	Eradication eliminated H. pylori, reduced activity and inflammation, improved corpus atrophy; no worsening of reflux or need for higher omeprazole dose
Schenk et al., 1998 [14]	Prospective comparative study	26 GERD patients on long-term omeprazole; 12 with severe hypergastrinemia (>400 ng/L) vs 14 with normogastrinemia (<300 ng/L); H. pylori serology performed	Omeprazole maintenance therapy, mean duration 55–80 months	Severe hypergastrinemia group vs normogastrinemia group	Gastrin levels, gastric inflammation, corpus atrophy, pepsinogen A, pepsinogen A/C ratio, mucosal somatostatin, gastric emptying, vagal nerve function	Approx. 4–6.5 years	Severe hypergastrinemia was associated with longer therapy duration and H. pylori infection. It correlated with corpus inflammation, atrophic gastritis, reduced pepsinogen and somatostatin, but was not linked to vagal dysfunction or delayed gastric emptying
Lundell et al., 1999 [15]	Randomized controlled trial (Nordic GERD Study Group)	310 GERD patients with esophagitis/chronic GERD; H. pylori status mixed (40 omeprazole group, 53 ARS group were positive)	Omeprazole maintenance therapy, long term (3 years)	Anti-reflux surgery (ARS)	Gastric glandular atrophy, corpus gastritis, intestinal metaplasia, serum gastrin	3 years	No significant difference between omeprazole and ARS groups. Progression of atrophy was slow and occurred irrespective of therapy. Intestinal metaplasia was rare (only type I) and not influenced by treatment. Basal gastrin higher in H. pylori positive, especially omeprazole users, but did not rise further during follow-up.
Uemura et al., 2000 [16]	Prospective clinical trial	141 H. pylori positive patients with peptic ulcers or reflux oesophagitis	Omeprazole 20 mg/day for 3 years (n=7) OR omeprazole 8 weeks → famotidine 40 mg/day maintenance (n=134)	Omeprazole long-term vs omeprazole short course then famotidine	H. pylori density, neutrophil infiltration, gastritis scores (antrum, corpus), atrophy, intestinal metaplasia	3 years	Famotidine group: decreased H. pylori density and inflammation in antrum, but not corpus. Corpus gastritis worsened in patients with mild baseline gastritis, improved in those with moderate/severe. Omeprazole-only group (n=7): reduction in inflammation in both antrum and corpus. No increase in intestinal metaplasia or atrophy observed.
Yang et al., 2009 [17]	Randomized controlled trial	325 reflux esophagitis patients: 105 H. pylori positive eradication, 105 H. pylori positive non-eradication, 115 H. pylori negative	Long-term esomeprazole; eradication group received 1-week triple therapy	H. pylori positive eradication vs non-eradication vs H. pylori negative controls	Gastric atrophy and intestinal metaplasia progression/regression	2 years	H. pylori negative group: no progression of atrophy/IM. Eradication group: regression of atrophy and IM (P<0.05). Non-eradicated group: increased prevalence and progression of atrophy and IM at 2 years. Eradication significantly reduced progression and promoted regression compared to non-eradicated controls (P<0.001).
Geboes et al., 2001 [18]	Prospective clinical trial	78 patients with endoscopically proven esophagitis; 34 H. pylori negative, 39 H. pylori positive (various baseline gastritis patterns)	Lansoprazole 30 mg daily, 5 years	H. pylori positive vs negative	Gastritis progression (antral vs corpus), atrophy, intestinal metaplasia	5 years	H. pylori negative: no histological changes over 5 years. H. pylori positive: progression to pangastritis in 20 patients, with reduced antral activity in most. No increase in intestinal metaplasia. Trend towards regression of antral atrophy but increase in corpus atrophy. Long-term lansoprazole effective clinically but mucosal changes depended on H. pylori status.

Quality Assessment

The quality assessment of the included studies, summarized in Table 2, demonstrates that overall risk of bias was either low or moderate, reflecting reasonable methodological robustness but with some limitations. Randomized controlled trials generally showed low risk, benefiting from appropriate randomization, balanced groups, and the use of standardized histological assessments such as the Sydney system. In contrast, prospective clinical and comparative studies were judged at moderate risk, primarily due to non-randomized allocation, small sample sizes, and potential baseline imbalances, particularly regarding H. pylori status. Attrition was present across several trials but was typically balanced and transparently reported, minimizing concerns of bias. Importantly, none of the studies exhibited evidence of selective reporting, and outcome measurements were consistently based on histology and validated laboratory methods. These findings suggest that while the evidence base is credible, the reliance on older, smaller, and non-randomized studies warrants cautious interpretation of results.

**Table 2:** The risk of bias assessment of included studies on long-term proton pump inhibitor therapy. RoB 2: Cochrane Risk of Bias 2 tool; ROBINS-I: Risk of Bias in Non-randomized Studies of Interventions; IM: Intestinal metaplasia; GERD: Gastroesophageal reflux disease

Study (Author et al., Year)	Study Design	Risk of Bias Tool	Randomization / Confounding	Deviations from Intended Interventions	Missing Data	Outcome Measurement	Selective Reporting	Overall Risk of Bias
Kuipers et al., 2004 [13]	Randomized controlled trial	RoB 2	Adequate randomization, groups balanced	No major deviations	Moderate attrition but balanced	Histology with Sydney system	No selective reporting	Low
Schenk et al., 1998 [14]	Prospective comparative study	ROBINS-I	Potential confounding (small sample, baseline H. pylori differences)	No deviations	Low attrition	Biopsies and labs, standardized	No selective reporting	Moderate
Lundell et al., 1999 [15]	Randomized controlled trial	RoB 2	Randomization appropriate (large sample)	No major deviations	Moderate attrition, well reported	Histology by Sydney system	No selective reporting	Low
Uemura et al., 2000 [16]	Prospective clinical trial	ROBINS-I	Allocation not randomized; small omeprazole-only subgroup	No deviations	Follow-up acceptable	Histology standardized	No selective reporting	Moderate
Yang et al., 2009 [17]	Randomized controlled trial	RoB 2	Randomization adequate	No deviations	Some attrition, similar across groups	Histology standardized	No selective reporting	Low
Geboes et al., 2001 [18]	Prospective clinical trial	ROBINS-I	H. pylori status not randomized; baseline gastritis variation	No deviations	Minimal attrition	Histology by biopsy, consistent	No selective reporting	Moderate

Discussion

This systematic review found that long-term proton pump inhibitor therapy does not uniformly accelerate the development of gastric atrophy or intestinal metaplasia, but the presence of Helicobacter pylori plays a critical modifying role. Across randomized and observational studies, patients without H. pylori generally showed stable histological findings during prolonged acid suppression, whereas those with persistent infection demonstrated progression to corpus-predominant gastritis and, in some cases, increased atrophy. Trials such as Kuipers et al. [13] and Yang et al. [17] consistently showed that eradication of H. pylori not only prevented progression but also induced regression of atrophy and intestinal metaplasia, underscoring the interplay between acid suppression and chronic infection. Collectively, these findings suggest that adverse mucosal changes are not an inevitable consequence of PPI use, but rather are contingent on microbial and host factors.

The results of this review align with earlier evidence suggesting that PPIs alone are not direct drivers of precancerous gastric changes, but that risk is amplified in the setting of H. pylori infection [19]. Previous reports, including long-term follow-ups in Western populations, similarly demonstrated that progression of atrophy was slow and not accelerated by acid suppression alone, consistent with the findings of Lundell et al. [15]. However, studies from East Asia, such as Uemura et al. [16], highlighted variable outcomes, likely reflecting higher baseline gastric cancer risk and differences in H. pylori virulence. The lack of increased intestinal metaplasia in Geboes et al. [18] contrasts with the significant regression reported by Yang et al. [17] following eradication, suggesting that population differences, duration of therapy, and biopsy site selection may explain discrepancies. Taken together, these comparisons reinforce that the risk of gastric atrophy during long-term PPI therapy is context-dependent, with H. pylori eradication emerging as the most consistent protective factor across geographic and methodological variations.

Taken together, these comparisons reinforce that the risk of gastric atrophy during long-term PPI therapy is context-dependent, with H. pylori eradication emerging as the most consistent protective factor across geographic and methodological variations. While the biological plausibility of acid suppression interacting with persistent H. pylori to promote atrophy is well supported [19-21], the real-world clinical significance appears more limited, as most trials demonstrated slow progression and low absolute rates of intestinal metaplasia over several years of follow-up [15-18].

The included studies demonstrate notable methodological strengths, particularly the use of randomized controlled trials such as Kuipers et al. [13], Lundell et al. [15], and Yang et al. [17], which provided robust comparative data with long-term follow-up and histological assessment based on the Sydney system. However, important limitations temper the certainty of conclusions. Some trials had small sample sizes, such as Schenk et al. [14] with only 26 participants, limiting generalizability. Non-randomized designs, including Uemura et al. [16] and Geboes et al. [18], introduced potential confounding, while attrition over multi-year follow-up may have introduced bias. Definitions of atrophy and intestinal metaplasia were not always standardized, and stratification by H. pylori status was inconsistently applied. Moreover, most of the evidence comes from the late 1990s and early 2000s, when clinical practice and available PPIs differed from today, raising questions about applicability to current therapeutic strategies with newer agents such as esomeprazole or rabeprazole.

The biological plausibility of the observed associations strengthens the interpretation of these findings. Long-term acid suppression by PPIs induces sustained hypergastrinemia, which may exert trophic effects on enterochromaffin-like cells and alter gastric mucosal physiology [20]. In patients with persistent H. pylori infection, acid suppression promotes a shift of gastritis towards the corpus, where chronic inflammation can accelerate glandular atrophy and intestinal metaplasia [21]. By contrast, in H. pylori-negative patients, such adverse histological progression is rarely observed, suggesting that the bacteria, rather than the PPI itself, is the primary driver of precancerous changes. These mechanistic insights explain why eradication of H. pylori consistently reduced or reversed atrophy in studies such as Kuipers et al. [13] and Yang et al. [17], highlighting the synergy between infection status and pharmacologic acid suppression.

From a clinical perspective, the findings suggest that long-term PPI therapy alone does not confer a high risk of atrophy or intestinal metaplasia, but the presence of H. pylori infection substantially increases vulnerability [22]. This supports the recommendation that patients who require prolonged PPI therapy should be screened and treated for H. pylori before initiating maintenance therapy. Importantly, PPIs remain highly effective and safe for conditions such as GERD, peptic ulcer disease, and Barrett’s esophagus, but their use should be periodically re-evaluated to avoid unnecessary prolonged therapy [23]. On a public health level, the evidence highlights the importance of integrating H. pylori management with acid suppression strategies to minimize long-term cancer risk. Future guidelines may need to emphasize both eradication protocols and careful surveillance of high-risk patients [24,25].

This review is limited by the relatively small number of available trials, most of which were conducted before 2005, when newer PPIs were not yet widely studied. The included studies were often geographically restricted to Europe and Asia, introducing regional biases in both H. pylori prevalence and gastric cancer risk. Sample sizes were small in several trials, and attrition was common in long-term follow-up. In addition, heterogeneity in biopsy sampling protocols, histological scoring, and definitions of atrophy and metaplasia makes direct comparison across studies challenging. The absence of contemporary randomized data on modern PPIs such as rabeprazole or esomeprazole further limits applicability to present-day clinical practice.

Future studies should focus on large-scale, multicenter randomized controlled trials with long-term follow-up, standardized histological endpoints, and consistent reporting of precancerous outcomes such as atrophy, intestinal metaplasia, polyps, and hypergastrinemia. The role of newer acid suppressants, including vonoprazan and potassium-competitive acid blockers, should be investigated to determine whether their pharmacodynamics alter gastric mucosal risk compared to conventional PPIs. Research should also evaluate surveillance strategies for patients requiring lifelong therapy, particularly those at high risk of gastric cancer. Finally, incorporation of molecular biomarkers, including serum pepsinogen, gastrin levels, chromogranin A, and genetic predisposition markers, may provide more precise risk stratification and improve preventive strategies.

## Conclusions

This review suggests that the potential risk of precancerous gastric changes may not stem directly from proton pump inhibitors themselves, but rather appears to be influenced by the presence or absence of Helicobacter pylori infection. Across the six included trials, eradication of H. pylori was consistently associated with stabilization or regression of gastric atrophy and intestinal metaplasia, whereas persistence of infection was linked to progression of mucosal damage. Although some studies reported hypergastrinemia and corpus atrophy during long-term therapy, these findings were inconsistent and context-dependent. Taken together, the evidence indicates that proton pump inhibitors remain safe and effective for prolonged use when appropriately prescribed, but their long-term impact on gastric mucosa is best understood in relation to H. pylori status. Future research should focus on large-scale, multicenter prospective trials that apply modern histological standards and standardized definitions of atrophy and intestinal metaplasia. Studies evaluating newer acid-suppressing agents, longer follow-up durations, and integration of molecular biomarkers may further clarify risks and refine strategies for cancer prevention in patients requiring sustained acid suppression.
